# Crosstalk between Lipid Rafts and Aging: New Frontiers for Delaying Aging

**DOI:** 10.14336/AD.2022.0116

**Published:** 2022-07-11

**Authors:** Shuo Zhang, Neng Zhu, Jia Gu, Hong-Fang Li, Yun Qiu, Duan-Fang Liao, Li Qin

**Affiliations:** ^1^Division of Stem Cell Regulation and Application, School of Pharmacy, Hunan University of Chinese Medicine, Changsha, China.; ^2^Department of Urology, The First Hospital of Hunan University of Chinese Medicine, Changsha, China.; ^3^Hunan Province Engineering Research Center of Bioactive Substance Discovery of Traditional Chinese Medicine, Hunan University of Chinese Medicine, Changsha, China.

**Keywords:** aging, senescence, lifespan, aging hallmarks, lipid raft, membrane raft

## Abstract

With the rapid aging in the global population, delay of aging has become a hot research topic. Lipid rafts (LRs) are microdomains in the plasma membrane that contain sphingolipids and cholesterol. Emerging evidence indicates an interesting interplay between LRs and aging. LRs and their components are altered with aging. Further, the aging process is strongly influenced by LRs. In recent years, LRs and their component signaling molecules have been recognized to affect aging by interfering with its hallmarks. Therefore, targeting LRs is a promising strategy to delay aging.

## 1. Introduction

The aging global population poses an increasing burden on public healthcare. In 2020, there were 722 million individuals aged over 65 years (accounting for 9.318 percent of the total population), and the proportion of senior people continues to rise (data from the World Bank: population ages 65 and above, total (accessed December 10, 2021) https://databank.shihang.org/home.aspx). The incidence of cardiovascular and cerebrovascular diseases, neurodegenerative diseases, metabolic diseases, and cancers with aging is also increasing each year.

Aging is generally considered a complicated process that cannot be avoided. However, it does have common denominators, such as 1) loss of proteostasis 2) altered intercellular communication 3) genomic instability 4) mitochondrial dysfunction 5) epigenetic alterations 6) deregulated nutrient-sensing 7) cellular senescence 8) telomere attrition 9) stem cell exhaustion [1]. Lipid rafts (LRs) are key functional microdomains involved in signal transduction and membrane trafficking. Because signal transduction is essential for aging processes, the relationship between LRs and aging has attracted increasing attention. Furthermore, recend studies indicate that LRs are promising targets to attenuate or delay human aging, which is signifcant for regulating aging procedures and achieving therapeutic effects.

## 2. Structure and functions of LRs

The LR hypothesis was proposed in 1997 [2]. LRs are microdomains (10-200 nm) with a short life, and their components, such as sphingolipids, cholesterol, and proteins, are assembled to function and disassemble quickly afterward. The components and sizes of LRs are not constant, and they can merge with each other to become larger when necessary [3]. As hubs for signal transduction, LRs contain various signal proteins, including glycosylphosphatidylinositol (GPI)-anchored proteins, Src family kinases (SFKs), and Epidermal growth factor receptor (EGFR) [4]. LRs are mainly composed of sphingolipids, which tend to display longer and more saturated hydrocarbon chains and contribute to thickening LRs. Moreover, sphingolipids are rich in oxygen-containing groups that can form hydrogen bonds, making LRs more tightly packed than the surrounding areas. Known as liquid-ordered domains (L_o_), these tightly packed regions are surrounded by a sea of liquid-disordered (L_d_) phospholipids that lack cholesterol [5]. Because sphingolipids are prone to forming hydrogen bonds, cholesterol has a slightly stronger affinity for sphingolipids. Specifically, cholesterol serves as a spacer and dynamic glue between hydrocarbon chains to assemble the LRs and maintain their integrity [6,7].

Caveolae are stereoscopic-type LRs that are invaginated into the plasma membrane; their proteins differ from those found in LRs and include the specific caveolin and cavin family members [8]. Among the extensively studied caveolin family proteins, caveolin-1 is a critical regulator of cell senescence [9-11].

Owing to the complexity of LR composition and their “small, heterogeneous, and highly dynamic” characteristics, their separation and visualization progress remain hindered to a certain extent. Despite this controversy, detergents such as Triton X-100 are still used to purify LRs [12]. Sucrose gradient centrifugation is the most used method for further fractionation of LRs [13]. Next, markers of LRs, such as sphingolipid/cholesterol [13], ganglioside M1(GM1) [14], CD36 [15], flotillin-1, and caveolin-2 [16], are identified by chromatography-mass spectroscopy or Western blotting to determine the LR fractions. Usually, LRs cannot be observed directly in living cells, but fluorescent probes are available for their imaging. For example, Alexa Flour 488/555/594-conjugated cholera toxin B (CtxB) can label GM1 in green/orange/red fluorescence to visualize LRs indirectly [17]; Laurdan staining, which emits blue fluorescence, can also be used to observe LRs [18,19]. In recent years, new fluorescent sphingomyelin analogs and fluorescent ganglioside analogs have been discovered, which facilitate LR tracking in living cells [20,21]; this has led to dramatic progress in the field.

**Figure 1.**
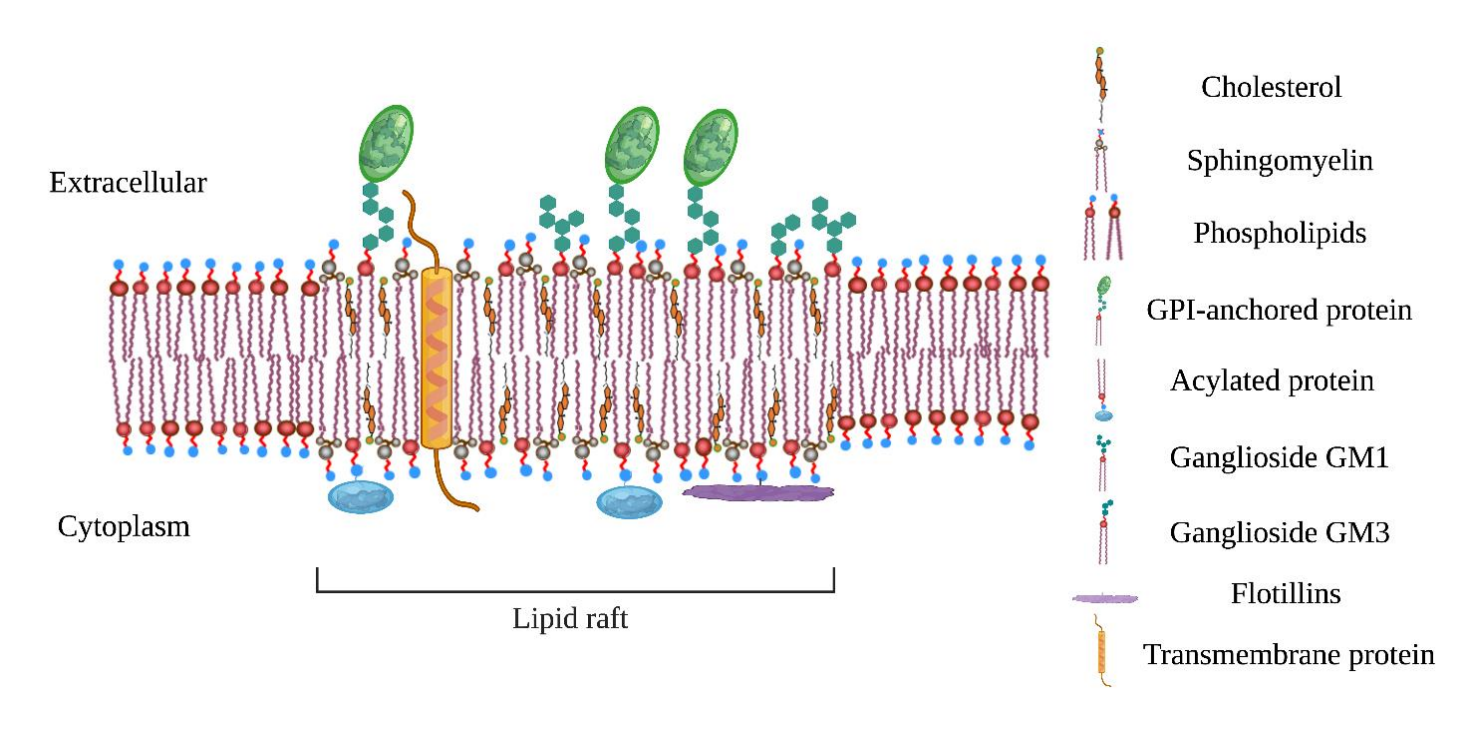
**Plane structure diagram of lipid rafts**. Constitutive LR residents include GM1, GM3, and GPI-anchored proteins on the outside of the plasma membrane, acylated proteins and flotillins inside the plasma membrane, and transmembrane proteins embedded in the plasma membrane.

## 3. Alterations of LRs during aging

The functions and composition of LRs are altered with aging. Such alterations occur in T-cells, neutrophils, fibroblasts, erythrocytes, and nerve cells. For instance, T cells from elder subjects have higher cholesterol and GM1 (a marker of LRs) levels in their LRs and lower LR fluidity [22,23]. In addition, the distribution of LRs is disorganized, whereas they are homogeneous in T-cells from young individuals [24]. This disorganization of the LR can reduce its aggregation, which may alter cellular signal transduction and communication. In aging human fibroblasts, LRs, cholesterol, and flotillin (an LR marker) are reduced [25], whereas polymerase I and transcript release factor (PTRF), a member of the cavin family in caveolae, is increased [26]. Further, signaling competent caveolae are lost, and the caveolae fraction contains lower levels of caveolin 1 and 2, resulting in impaired signal transduction [27]. In human red blood cells, the LR protein marker flotillin-2 also decreases during aging [28]. Likewise, in human frontal cortex nerve cells, LR structures are substantially altered when the brain cortex ages, which is termed “LR aging” [29]. Regarding LR functions, these microdomains are known to be involved in the initial signal complex formation during T cell activation [30]. With increasing age, LRs recruit less lymphocyte-specific protein tyrosine kinase (Lck) and linker of activated T cells (LAT) (T cell regulators [31-33]) causing compromised T cell function in seniors [22]. In neutrophils, the LR-relevant Toll-like receptors 4 (TLR4) signaling pathway is altered with aging. Under lipopolysaccharide (LPS, a TLR4 ligand) stimulation, there is no recruitment of the TLR4 downstream signal molecule IL-1 receptor-associated kinase-1 (IRAK-1) to LRs in elderly donor neutrophils, which results in impaired TLR4-driven signaling events [34]. Changes in the role of LRs with aging could thus be a cause of decreased neutrophil function. Owing to the dysfunction of T cells and neutrophils during aging, the immune response is reduced, contributing to immunosenescence. Notably, a study published in Nature has indicated that immunosenescence drives the aging of other organs, ultimately promoting organism aging [35].

Similar results have also been reported in animals. In wild-type mice, LRs undergo age-related changes, such as decreased cholesterol content and increased sphingomyelin levels [36]. In mouse CD8+ T cells, GM1 levels in LRs increase with aging [37]. However, there are no apparent age-dependent disparities in the GM1 levels of rat brain synaptic LRs. In addition, the content and activity of Ca^2+^-ATPase (an intracellular free Ca^2+^ precise regulator) in the LR domain is downregulated with increasing age, and importantly, Ca^2+^ homeostasis dysregulation is associated with brain aging [38].

Together, these data emphasize that major changes occur in LR structure and function with age, suggesting that these changes may contribute to cell signal transduction failure.

**Table 1:** Alterations in lipid rafts and their composition with aging.

Species	Notes	Age-related alteration in LRs	Age-related alteration in components of LRs	References
**Human**	T cells	The fluidity of LRs ↓	Cholesterol ↑	[22]
		The distribution of LRs is disorganized	GM1 ganglioside ↑	[22,23]
	Fibroblasts	The signaling competent caveolae ↓	Cholesterol ↓	[25,27]
			Flotillin ↓	[25]
			PTRF ↑	[26]
	Neutrophils	The LR-dependent TLR4 signal ↓	TLR4 ↑	[34]
	Red blood cells		Flotillin-2 ↓	[28]
	Nerve cells	The lipid structure (phospholipid-bound fatty acids and specific lipid classes) of LRs is altered		[29]
**Mouse**	Frontal lobe		Cholesterol ↓	[36]
			Sterol ester ↓	[36]
			Sphingomyelin ↑	[36]
			Saturated fatty acid ↑	[36]
			Phospholipids/cholesterol ratio ↑	[36]
	Cortical(3xTgAD)	LRs density ↑		[39]
	CD8 + T cell		GM1 ganglioside ↑	[37]
**Rat**	Cerebral synaptic cells		GM2 ganglioside -	[38]
			Ca^2+^ -ATPase protein ↓	[38]
**Rhesus macaque**	Frontal lobe		GM3 ↑	[40]
			Sphingomyelin ↑	[40]

-: invariant; ↓: decrease; ↑: increase; 3xTgAD: a triple-transgenic model of Alzheimer's disease

## 4. LR and genomic instability

Accumulation of genetic damage is a generally recognized cause of genomic instability [41], which includes direct lesions in DNA (nuclear DNA damage, mitochondrial DNA damage, telomere attrition) as well as defects in nuclear architecture [1]. Usually, organisms can repair themselves after DNA damage, but severe DNA damage or a lack of DNA repair exacerbates the aging process [1,42,43].

LRs can affect DNA integrity. For example, LR-mediated signaling can modulate reactive oxygen species (ROS) to influence DNA damage and repair responses, and LR disruption can suppress DNA repair responses [44]. Meanwhile, deficiency of CD59 (a GPI-anchored protein on LR) can exacerbate DNA damage and induce cellular senescence [45]. The level of caveolin-1 in LRs is often upregulated after DNA damage and this activates DNA repair [46].

These results indicate that genomic stability can be affected by LRs and the relevant signals to ameliorate aging.

## 5. LR and loss of proteostasis

Proteostasis can control non-native proteins accumulate through molecular chaperones, cochaperones, and proteolytic systems [47]. However, proteostasis diminishes with age [48], enhancing the risk of protein misfolding and aggregation, a hallmark of aging [1], which may be deleterious to cells [47,49]. Notably, LRs are integral to proteostasis.

Molecular chaperones, heat shock protein (HSPs), assist protein refolding [50]. According to previous reports, distinct reorganization of LRs is required to generate and transmit stress signals for stimulating HSP genes, thereby upregulating HSP expression [51]. Further studies have revealed that this reorganization is induced by Ras-related C3 botulinum toxin substrate 1 (Rac1)-mediated actin polymerization [52,53]. Meanwhile, the level of heat-induced HSP expression is impaired if LRs are disrupted [54]. These results indicate that the signal for HSP gene activation is transmitted through LRs. Although the role of LRs remains unclear, evidence suggests that remodeling plasma LRs can activate stress signal transduction pathways [55]. Notably, lifespan is positively determined by HSPs, and HSP expression has been shown to extend the lifespan of Drosophila [56]. HSP induction during aging may thus preserve protein homeostasis and lifespan by refolding damaged proteins that accumulate throughout aging.

If these folding attempts are futile, abnormal proteins are degraded by two central proteolytic systems (ubiquitin/proteasome and autophagic/lysosomal systems), which also decay with age [57]. Epidermal growth factor (EGF) signaling can affect C. *elegans* longevity by stimulating the ubiquitin/proteasome system [58]. Meanwhile, the EGF receptor is localized to LRs [59], which means that ubiquitin/proteasome system activity can be enhanced by activating EGFR on LRs to help maintain protein homeostasis and prolong lifespan. Further, the autophagic/lysosomal system is linked with mammalian target of rapamycin (mTOR) activation; specifically, autophagy can be stimulated by mTOR downregulation [60]. LRs appear to be essential for regulating the mTOR pathway by promoting phosphoinositide 3-kinase (PI3K) recruitment and V-akt murine thymoma viral oncogene homolog (Akt) activation [61,62]. Moreover, phosphatase and tensin homologue protein (PTEN) can suppress the PTEN/Akt/mTORC1 pathway, thereby activating autophagy by mobilizing the LR domain [63,64]. As an integral component of LRs, cholesterol is another factor that affects autophagy. Cholesterol accumulation reduces autophagic activity by suppressing the fusion of lysosomes with autophagic vacuoles [65,66]. Notably, autophagy has a beneficial systemic effect on lifespan [67].

As previously discussed, LR remodeling favors the activation of HSPs, thereby refolding deleterious proteins. Further, excitation of the ubiquitin/proteasome system and autophagic recovery of protein homeostasis provide exciting possibilities for extending longevity.

## 6. LR and deregulated nutrient-sensing

Deregulated nutrient sensing is considered a hallmark of aging. It is closely affiliated with several nutrient-sensing systems, such as the insulin/insulin-like signaling pathway (IIS) pathway, which is involved in glucose sensing; mTOR, which participates in the detection of elevated amounts of amino acids [1,68]. Nutrient-sensing system alterations affect lifespan as increased nutrient signaling speeds up aging whereas reduced nutrient signaling prolongs lifespan, that is , reduces the functions of growth hormone (GH), insulin-like growth factor 1 receptor (IGF-1R), or downstream biological factors such as Akt and mTOR, and increases the activity of AMPK, Sirt1, PTEN, and Forkhead box class O (FOXO) [1,69]. Signal transduction and activation of the nutrient-sensing systems mentioned above require the participation of LRs.

The IIS pathway is regulated by LRs in multifaceted ways. First, activation of the IIS signal requires ligands to bind to the central regulator IGF-1R (located in LRs) [70]. Second, LRs are indispensable for IGF-1R downstream signals [70]. The binding of IGF-1 and IGF-1R activates the PI3K/Akt pathway, and the phosphorylation of Akt requires LRs. If LRs are destroyed, Akt phosphorylation is blocked [71], possibly because Akt activation requires PI3K recruitment to LRs [62]. Akt has many downstream targets including mTOR and FOXO. Among these, FOXOs represent a well-conserved group of transcription factors; however, when phosphorylated, they lose their ability to function as transcriptional activators [72,73]. Inhibition of LR clustering has been reported to impede the PI3K/ Akt/ FOXO pathway [74]; in particular, FOXO phosphorylation is attenuated by LR disruption [75], suggesting that LRs are involved in FOXO signal transduction. Additionally, FOXO is a core longevity-promoting transcription factor involved in the IIS pathway [76,77]. Upregulating FOXO activity through the regulation of LRs is thus a promising way to delay aging [76].

CD24, located in the LR, can recruit PTEN to the LR and modulate the downstream pathway [63]. Upon PTEN inhibition, the PI3K/Akt signaling pathway is activated and upregulates mTOR1 [78], thereby interfering with protein and lipid synthesis as well as energy metabolism [79].

In summary, LR can mobilize and activate IIS pathway signaling molecules, which in turn regulate the signal transduction of mTOR and FOXO. Thus, prolonging health span by controlling LR is theoretically feasible.

## 7. LR and mitochondrial dysfunction

Mitochondrial dysfunction leads to accelerated aging in mammals [1,80-82]. Recently, LRs and their residents have been reported to modulate mitochondrial function. Data from Yu’s laboratory revealed that caveolin-1 deficiency limits the expression of cardiolipin biosynthetic enzymes that decrease cardiolipin content (an essential lipid for mitochondrial respiration), thereby reducing mitochondrial respiration, culminating in mitochondrial dysfunction and premature senescence [83]. Asterholm and colleagues found caveolin-1-null mouse embryonic fibroblasts displayed altered mitochondrial metabolism and higher mitochondrial membrane potential [73]. Furthermore, caveolin-1 deficiency leads to mitochondrial dysfunction by reducing membrane fluidity and mitochondrial respiratory chain efficiency, consequently causing ROS buildup [84]. Actually, the LR has more than one molecule involved in mitochondrial function. Src kinases, one of LR residents, are also important regulators of mitochondrial function, and have emerged as key players in mitochondrial tyrosine phosphorylation events [85]. Inhibition of SFKs ameliorates mitochondrial dysfunction [86]. Hunterour *et al.* discovered that c-Src, one of the most prevalent SFKs undermines mitochondrial energy metabolism by weakening the mitochondrial oxidative phosphorylation complexes [87]. Cholesterol also affects mitochondrial function as oxidized cholesterol derivatives (7-ketocholesterol) impede mitochondrial metabolism by lowering membrane potential [88,89]. In particular, 7-ketocholesterol can modify cytoplasmic mitochondrial distribution and clusters [90].

The free radical theory of aging suggests that excessive ROS production by mitochondria damages the mitochondrial genome and proteins, causing deterioration of mitochondrial function as well as further organism dysfunction and shortens lifespan [91]. Aggregation of LR with nicotinamide adenine dinucleotide phosphate (NADPH) oxidase enzymes facilitates ROS production in intestinal epithelial cells [92]. Moreover, 7-ketocholesterol has been proved to interacts with NADPH-oxidase to trigger ROS overproduction [93-95]. Nuclear factor erythroid-2-related factor-2 (Nrf2), a leucine zipper transcription factor, can protect against harmful ROS and mediate the translation of antioxidant enzymes [96,97]. Here, caveolin-1 can restrict antioxidant enzyme expression by inhibiting Nrf2 endogenously [98] and accelerating premature senescence [99]. Thioredoxin reductase 1 (TrxR1) is a small oxidoreductase that contributes to the regulation of cellular redox homeostasis [100]. As it is also a caveolae resident protein, the combination of caveolin-1 and TrxR1 can inhibit TrxR activity, thereby accelerating stress-induced premature senescence [101].

Overall, Src kinases and caveolin-1 are key regulators of mitochondrial function in LRs and derepressing the abnormal activity of Src kinases or upregulating caveolin-1 is a promising strategy to ameliorate aging.

## 8. LR and cellular senescence

Generally, aging begins at the cellular level. Senescent cells accumulate as people grow older, resulting in aging and the promotion of age-related pathologies. However, mounting evidence has shown that LRs and their molecular composition are crucial for cellular senescence.

Stable cell cycle arrest is an important hallmark of cellular senescence [102]. Caveolin-1 can mediate cell cycle arrest, implying that caveolin-1 causes cellular senescence [103]. Furthermore, experimental results from Volonte et al. indicate that increased caveolin-1 expression causes senescence in murine fibroblasts, and that restoring caveolin-1 can reverse this condition [104].

With an in-depth molecular mechanism study based on phenotype, caveolin-1 has been shown to modulate senescence and organismal aging by inhibiting the effects of mouse double minute 2 homolog (Mdm2), protein phosphatase 2A-C subunit (PP2A-C), Sirt1, TrxR1, Nrf2, and EGFR on P53 [105]. First, caveolin-1 inhibits P53 degradation by binding Mdm2, followed by p53/p21 upregulation and induction of premature senescence [106]. Second, caveolin-1 triggers Ataxia telangiectasia-mutated (ATM) (P53 activator [107]) by isolating PP2A-C (ATM negative regulator) into caveolae domains, sequentially stimulating the p53/p21 pathway, leading to lung fibroblast senescence [108]. Third, caveolin-1 is a new Sirt1 blocker. The combination of Sirt1 and caveolin-1 caused by oxidants suppresses Sirt1 activity, which promotes p53 acetylation and induces premature senescence [109]. Serving as a TrxR1 antagonist, caveolin-1 suppresses TrxR activity, inhibiting the p53/p21 pathway, thus promoting premature senescence [101]. Furthermore, caveolin-1 can directly bind Nrf2 and prevent oxidant-induced Nrf2-related signaling, thereby accelerating aging [110]. Finally, caveolin-1 can directly combine with EGFR and limit its activation [111,112], thus attenuating EGF signaling in senescent cells [113]. Reducing caveolin-1 levels can restore the downstream signaling cascades of EGF cell cycle progression and reverse senescent phenotypes [114]. Growth factor responsiveness decreases because of the upregulated caveolin levels in senescent cells. Moreover, PTRF is necessary for caveolae to form and function [115,116] and is upregulated in senescent cells. Upregulated PTRF interacts with caveolin-1, leading to cellular senescence *via* the p53/p21 pathways [26,117].

In addition, 7-ketocholesterol was found to induce senescence in mouse endothelial progenitor cells *via* the Notch pathway [118]. 7-ketocholesterol has potential as an aging biomarker, as its accumulation is directly linked with various aging-related diseases [119,120].

Taken together, suppression of caveolin-1 or PTRF can clearly decelerate cellular senescence. On one hand, inhibiting caveolin-1 reverses the senescence phenotype [114,121]; on the other hand, reducing PTRF expression extends the cellular replicative lifespan [26]. However, cellular senescence is a terminal cell fate that prevents cells from proliferating indefinitely and can thus suppress tumorigenesis [122]. Therefore, determining the break-even point of cellular senescence is also a challenge in the future.

## 9. LR and stem cell exhaustion

Stem cells have been shown to replenish cells and regulate lifespan [123]. Stem cell exhaustion results in a decline in tissue regeneration, which is a significant hallmark of aging [1]. Stem cell surface molecules or secreted molecules from stem cells trigger hibernation or cell cycle entry [124,125]. Previous studies have shown that stem cell signaling pathways require LR to accurately modulate signal intensity [126].

LRs regulate stem cells in several ways. More specifically, LRs are indispensable for hematopoietic stem and progenitor cell (HSPC) retention in bone marrow niches and are hampered when LRs are disrupted [127]. The clustering of LRs in hematopoietic stem cells (HSCs) can augment downstream signaling pathways and deliver signals to cells, thereby inducing HSCs to re-enter the cell cycle. In contrast, the inhibition of LR aggregation disturbs PI3K/Akt/FOXO, resulting in the accumulation of FOXO transcription factors and expression of p57 cyclin-dependent kinase inhibitors, which cause HSC hibernation [74]. Additionally, LRs are required for the aforementioned pathway to be effectively activated, for recruiting the proliferation mediator Kit [128]. The mTOR signal is also mediated by LR *via* the PI3K/Akt pathway, and mTOR activity increases in HSCs with aging [129]. Using the mTOR inhibitor rapamycin to reduce mTOR activity can restore HSC function and increase lifespan [129]. However, the hematological toxicity of rapamycin cannot be ignored [130].

Intriguingly, LRs also function through LR-related proteins. Incorporation of CXC chemokine receptor 4 (CXCR4) into LRs activates Rac1 (a small GTPase involved in HSPC migration) and enables a more effective response to the stromal-derived factor-1 (SDF-1) gradient, which primes homing-related responses [131]. Further, the raft-resident protein Lyn (a tyrosine kinase belonging to the Src family) contributes to stem cell regulation [132]. In addition, Prion protein (PrP), a GPI-anchored protein mainly located in LRs [133], can stimulate mesenchymal stem cells [134] as well as HSCs [135] to proliferate and self-renew. LR-associated ADAM12 plays a pivotal role in esenchymal stem cell differentiation into smooth muscle cells [136].

Overall, LR clusters and LR-associated proteins have been implicated in stem cell regulation and the control of cell fate decisions. Treatments targeting LRs may thus be a new approach for stem cell rejuvenation.

## 10. LR and altered intercellular communication: inflammaging

Communication between cells is necessary for optimal collaboration; however, aging alters intercellular communication, including neuroendocrine dysfunction, inflammation, immunosenescence, and bystander effects [1]. Inflammation is a prevalent age-related alteration in intercellular communication [137]. The nuclear factor kappa-B (NF-κB) signaling pathway is a prominent inflammatory signaling pathway that regulates cellular inflammatory responses [138]. As the “ears and mouth” of cells, LRs are responsible for cellular signal transduction, especially inflammatory signaling. NF-κB signaling can be triggered by TLR and tumor necrosis factor alpha (TNF-α) receptors through binding to their corresponding ligands [139,140].

TLRs are transmembrane proteins of the pattern recognition receptor family. They can activate the pro-inflammatory NF-κB signaling pathway by binding to endogenous ligands [139]. TLR activation occurs in LRs. One study demonstrated that increased LRs recruit more myeloid differential protein-88 (MyD88)-dependent TLRs to LRs, boosting downstream signal transduction and promoting inflammation [141]. Moreover, TLR signal transduction requires the cooperation of raft protein [142]. CD14, a GPI-anchored protein localized in LRs [143], facilitates the transfer of LPS to TLR4 and subsequently activates the NF-κB pathway [144].

TNF-α, a proinflammatory cytokine, can activate NF-κB signaling and initiate an inflammatory response [145]. After the binding of TNF receptor 1 and TNFα, TNF receptor 1 translocates to LRs and initiates the transcription factor NF-κB by forming a receptor-induced signaling complex that binds several signaling proteins [140]. During this process, LRs act as a platform for TNF-α to mediate signal transduction. When LRs increase, TNF-α secretion is accelerated [146]. In senescent endothelial cells, caveolae and caveolin-1 are increased, whereas NF-κB activation induced by TNFα is decreased. Interestingly, this phenomenon can be reversed when caveolin-1 is knocked down. In other words, increased caveolae and caveolin-1 may inhibit the NF-κB pathway and prevent inflammation in senescent cells [147]. Meanwhile, 7-ketocholesterol induces TNF-α expression in human monocytes [148], indicating that it can affect cell communication.

Overall, LRs affect inflammation by regulating NF-κB expression. Numerous studies have focused on LRs to control the inflammatory status. For example, allicin inhibits mastitis by diminishing the LR form and inhibiting signals downstream of TLR2 and TLR6 [149]. Selenium is also used to alleviate lipopolysaccharide-induced endometritis by attenuating LR levels and impeding the recruitment of TLR4 into LRs [150]. Meanwhile, inhibiting NF-κB signaling is reported to delay senescence and aging in mice [151]. Hence, selectively blocking LR-dependent inflammatory processes may be a suitable strategy to delay aging.

**Figure 2.**
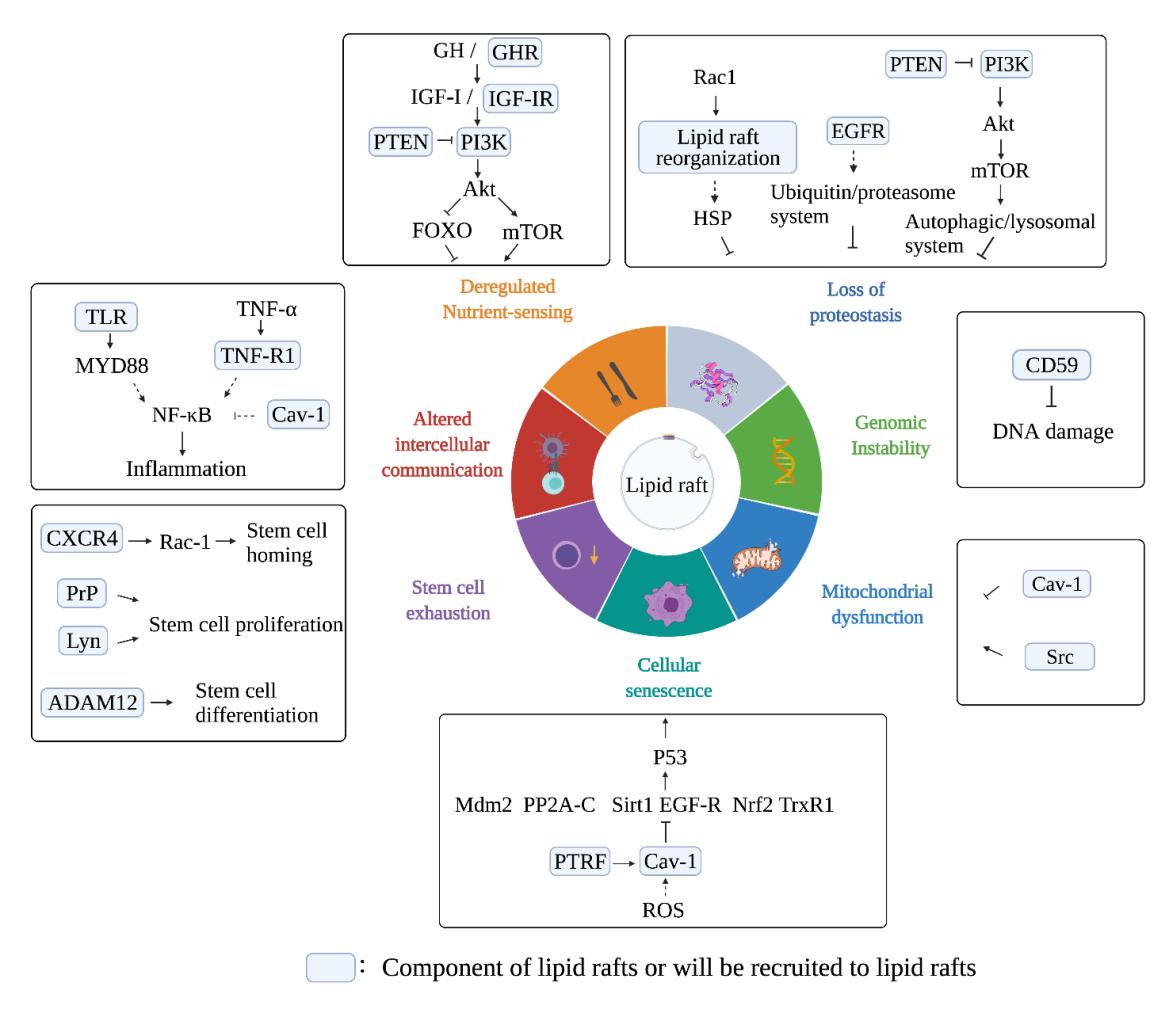
**Interplay between lipid rafts and aging hallmarks**. This figure shows the signaling pathways and molecules related to LR and the seven aging hallmarks described in this review.

## 11. Perspectives

The pursuit of longevity is the ultimate goal of humans, and aging remains a considerable challenge. Despite the continuous advances in our understanding of LRs over the last two decades, some questions remain unanswered. However, aging is apparently regulated by LR. Aging drastically affects the components and functions of LRs. Further, considering the evidence discussed here, the influences of LRs on the hallmarks of aging are apparent (Fig. 2). Many of these hallmarks contribute to the development of sustained inflammatory stage and aging [152]. Hence, attempts to “cure” aging should involve amelioration of inflammaging (chronic, sterile, low-grade inflammation during aging) [153], which can be achieved by regulating LRs.

Modulation of cholesterol is one way to regulate LRs, as cholesterol is a critical constituent of LRs. Most cellular cholesterol exists in the membrane and is enriched in LRs [154]. Depleting cholesterol can disrupt the form of LRs and reduce the content of LRs [149,155], suggesting that cholesterol-lowering drugs such as statins, can alleviate inflammaging to anti-aging by inhibiting the formation of LRs. As expected, clinical results have demonstrated that new statin use is associated with a decreased death rate among American veterans (75 years and older) [156]. However, one of the frequently reported adverse reactions of statins is memory impairment and cognitive decline [157,158]. Coincidentally, Alzheimer's disease, which is characterized by cognitive and memory deterioration, is associated with reduced levels of cholesterol and LRs in the frontal cortex [36,159]. Based on these results, we speculate that the adverse effects of statins on memory and cognitive alterations may partly be due to their cholesterol-lowering effects and hindered formation of LRs. Therefore, when using statins to delay aging, it is recommended to adopt some pharmaceutical modifications to increase the polarity of the statins or to choose hydrophilic statins instead of lipophilic statins for making them selective and inaccessible to the central nervous system, thus reducing their side effects.

Overall, aging has been proven modifiable, and some drugs for slow aging have been discovered. For example, rapamycin inhibits mTOR activation to delay aging; senolytics can target and eliminate senescent cells; sirtuin activators, which enhance sirtuin activity; Nicotinamide adenine dinucleotide (NAD) precursors that can supply cellular NAD levels; antidiabetic drugs such as metformin and acarbose; and non-steroidal anti-inflammatory drugs, can also be used [152,160]. However, none of drugs target LRs to delay aging, making it a future objective. Overall, targeting LRs will be a novel strategy for prolonging life, and statins might be promising candidates for new anti-aging agents.
